# Hip bone osteonecrosis with intraosseous pneumatosis after abdominal aortic aneurysm repair: a case of emphysematous osteomyelitis

**DOI:** 10.1259/bjrcr.20200138

**Published:** 2020-10-22

**Authors:** Amjad Chamsi Basha, Mohamed AbdelGhafour Khalifa, Fahad Albadr, Jamal Kaid, Hussein Alsakkaf

**Affiliations:** 1 College of Medicine, Sulaiman AlRajhi University PO Box 777, Al Bukairiyah, Saudi Arabia; 2 Department of Radiology, College of Medicine, King Saud University PO Box 145111, Riyadh, Saudi Arabia; 3 King Saud University Medical City, Department of Radiology, PO Box 2925, Riyadh, Saudi Arabia

## Abstract

Intraosseous pneumatosis is a rare and often fatal condition characterised by air accumulation in the bone that may be brought about by infection, trauma (surgical or otherwise), degenerative disease or neoplastic processes. Here, we present a case of pelvic emphysematous osteomyelitis following repair of an infected abdominal aortic aneurysm.

A 56-year-old Saudi male, known to have diabetes and hypertension, presented to the emergency department complaining of intermittent abdominal pain over the right lower quadrant. The patient was later diagnosed intraoperatively with an infected abdominal aortic aneurysm and treated appropriately. During multiple follow-up imaging studies, the patient was noted to have multiple intra-abdominal fluid collections, as well as intraosseous pneumatosis in the pelvis and right femur. 3 months later, intervention was again required due to patient deterioration and possible aortic graft leakage. Graft abscess was diagnosed and managed.

We present a case of an infected abdominal aortic aneurysm that eventually led to emphysematous osteomyelitis of the pelvis. This case report sheds light on intraosseous pneumatosis and emphysematous osteomyelitis, which is characterised by the former, in addition to signs of an underlying infection or abscess formation.

## Introduction

Intraosseous gas was first described as a characteristic of emphysematous osteomyelitis (EO) in the literature around 40 years ago.^1^ Intraosseous pneumatosis is often reported following trauma (iatrogenic or otherwise), fractures, and degenerative disease.^2,3^ To our knowledge, only 38 cases of EO have been reported in the literature to date. Although EO is a rare occurrence, it must be investigated and managed appropriately when it does occur. Here, we report the case of a 56-year-old male with EO of the pelvis following mycotic abdominal aortic aneurysm repair and provide a review of similar cases in the current literature.

## Case presentation

**Figure 1.**
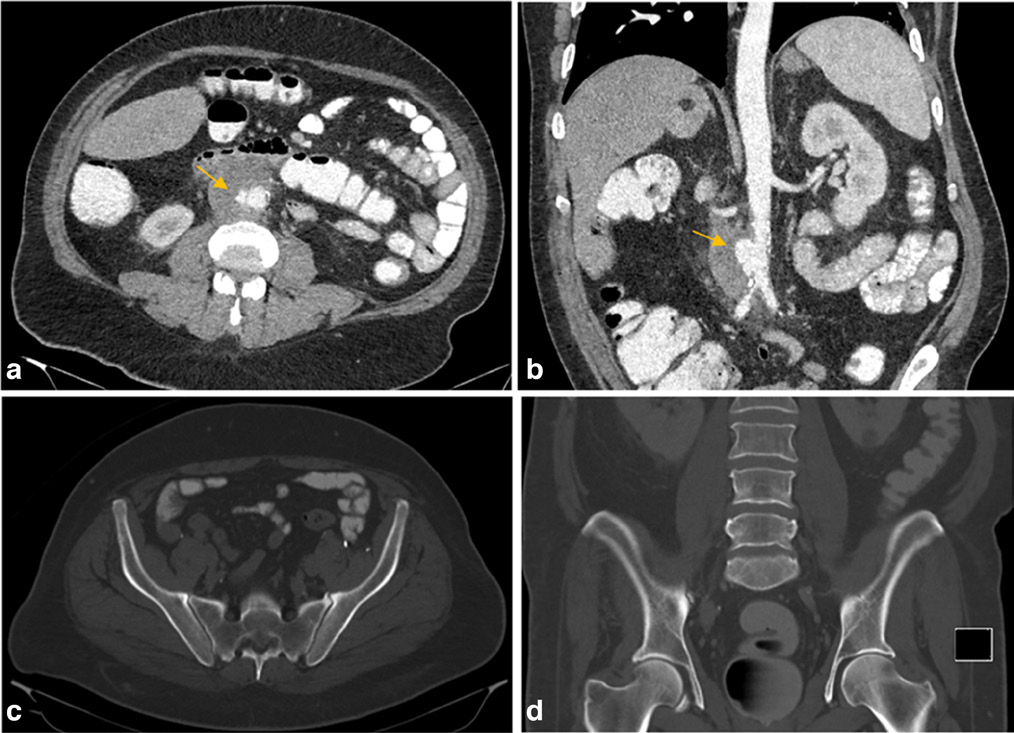
Initial CT with intravenous and oral contrast axial (a) and coronal (b) sections of the abdomen showing infrarenal aortic aneurysm with periaortic fat stranding (arrows). Axial (c) and coronal (d) bone window images show no intraosseous gas in the pelvic bones at the time of initial presentation.

A 56-year-old Saudi male with a 15 year history of insulin-dependent, poorly controlled diabetes mellitus Type 2 and a 10 year history of uncontrolled hypertension, presented to the emergency department complaining of intermittent right lower quadrant abdominal pain. The pain radiated to the back, moderate to severe in intensity, colicky in nature, and associated with nausea and vomiting. After a double-contrast abdominal CT scan was obtained, the patient was admitted with a provisional diagnosis of an infected infra renal abdominal aortic aneurysm (Figure 1). Open repair was performed with a Dacron graft. The patient developed ischaemic acute renal tubular necrosis and transient paraplegia below the T12 level following repair.

**Figure 2.**
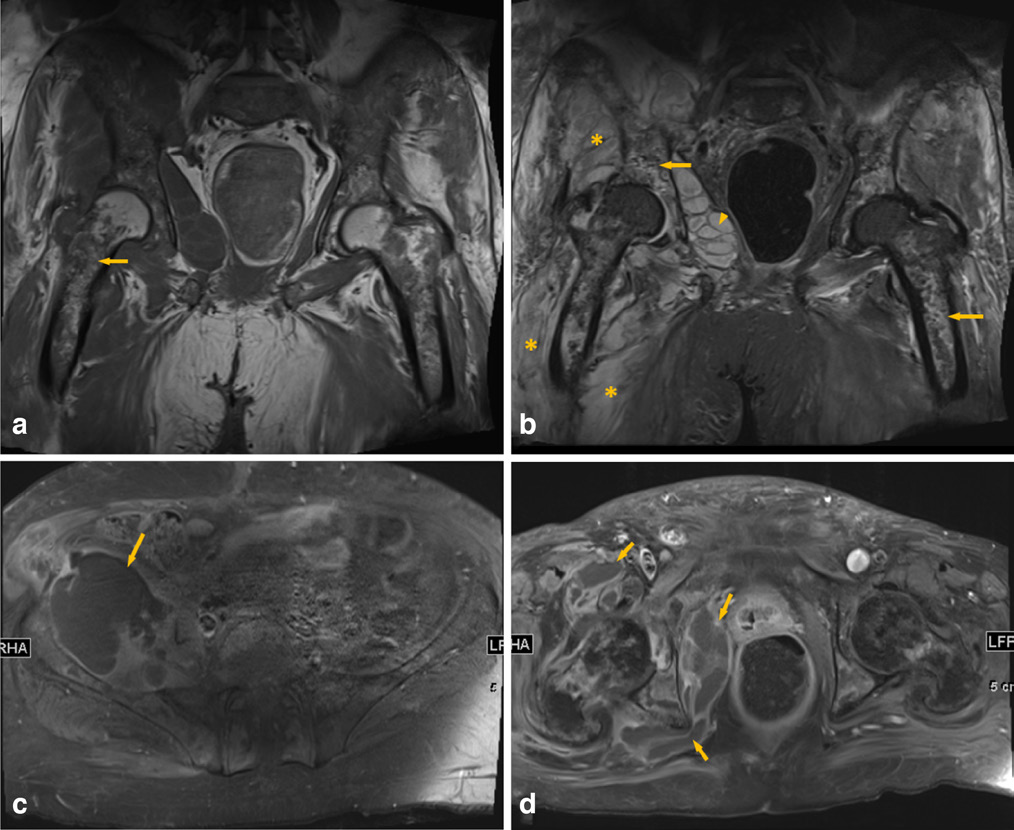
Around 1 month after initial presentation, coronal T1 (a) and fat saturated T2 (b) MRI of the pelvis show diffuse altered bone marrow signal intensity with areas of serpiginous low signal in T1 and high signal in T2 involving the pelvic and femoral bones, bilaterally indicating bone infarction/osteonecrosis (arrows). Associated multiple pelvic multiloculated fluid collections (arrowhead), as well as diffuse altered signal intensity of the pelvic muscles indicating myositis (asterisk) can be visualized as well. On axial post-contrast MRI (c, d), multiple pelvic collections are noted, the largest of which is in the right iliac crest muscle and around the left tuberosity (arrows).

Around 1 month later, the patient developed pelvic pain. CT scan of the abdomen showed a stable periaortic collection post-repair. MRI of the pelvis showed signs of osteonecrosis, as well as multiple large fluid collections in the right pelvis and around the ischial tuberosity as well as intraosseous collections (Figure 2), with compression of the right sciatic nerve. It was decided that the patient was to continue intravenous antibiotics without any surgical intervention.

**Figure 3.**
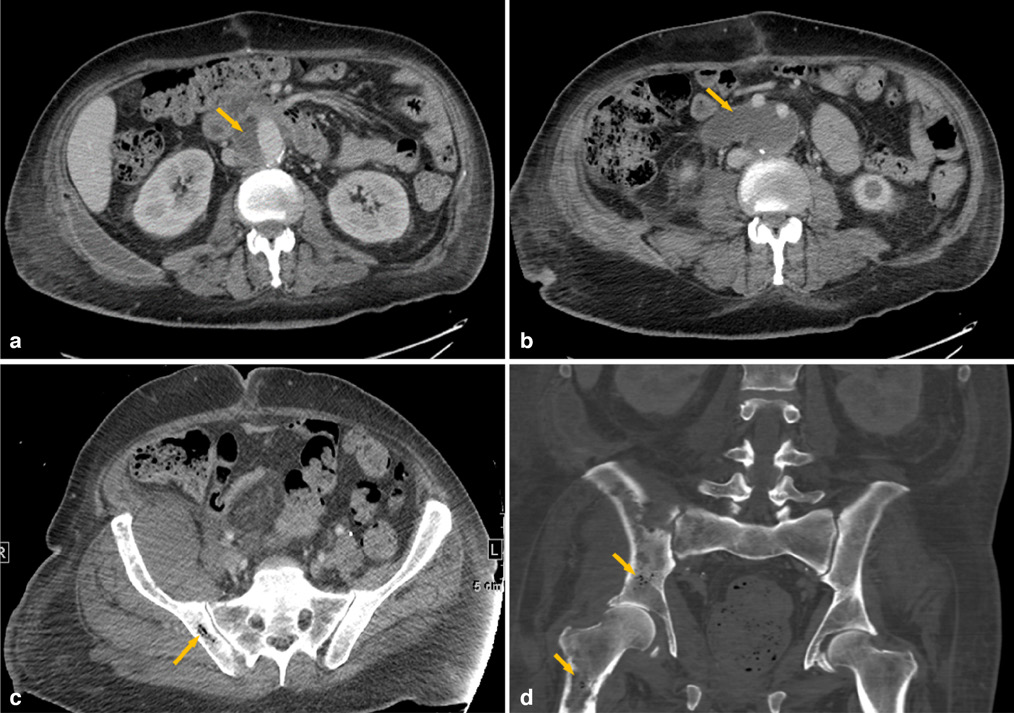
Post-contrast axial CT scan of the abdomen (a, b) showing periaortic fluid collections surrounding the infrarenal aorta down to the aortic bifurcation (arrows). Intraosseous pneumatosis with the ‘pumice stone’ sign typical of EO and bony lytic changes are seen in the right-sided pelvic bones and the proximal right femur (c, d). The right iliac crest muscle is swollen with surrounding fat stranding, corresponding with the findings seen on MRI in Figure 2. EO, emphysematous osteomyelitis.

**Figure 4.**
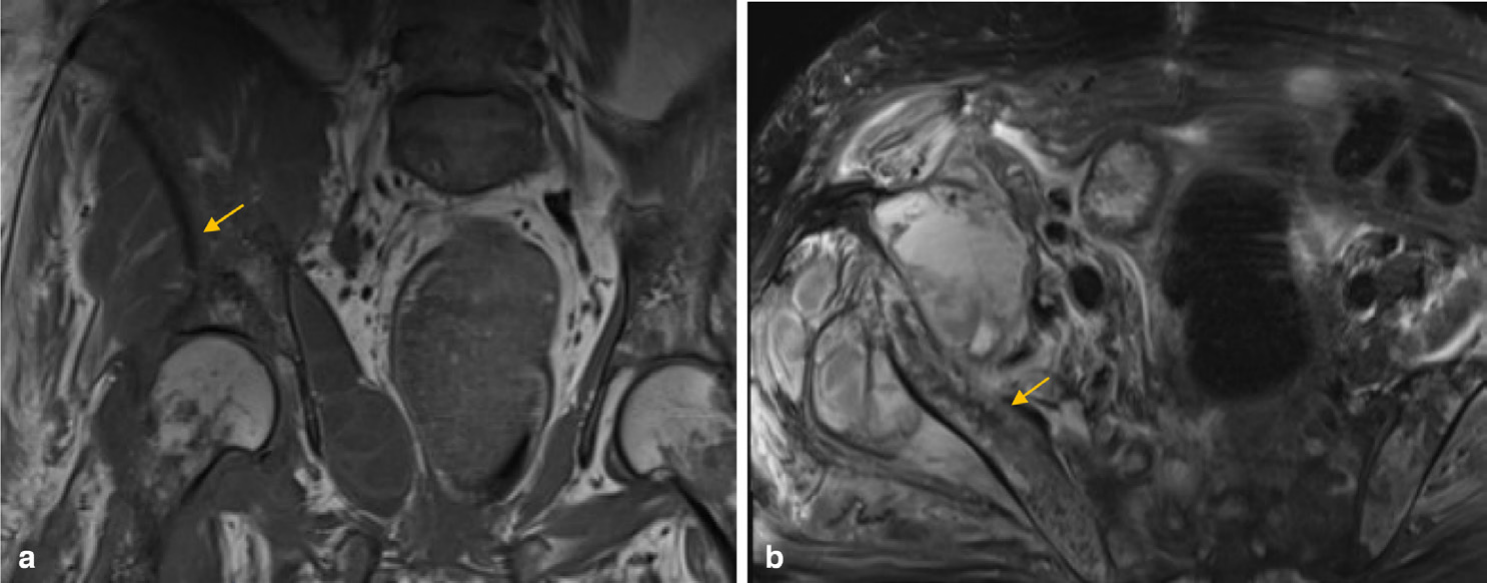
Previous coronal T1 (a) and axial fat saturated T2 (b) weighted images of the pelvis indicate multiple small T1 and T2 hypointense foci (arrows), corresponding to the intraosseous gas seen on CT scan.

Upon follow-up imaging, multiple stable loculated periaortic collections were detected surrounding the infra renal aorta and iliac bifurcation extending down to the pelvis, the largest of which measured 7 × 5 cm. Additionally, CT of the abdomen and pelvis showed small pockets of abscesses mainly involving the right iliacus, gluteus minimus, obturator internus, piriformis, and posterolateral and lower anterior abdominal wall muscles. More importantly, the CT scan showed intraosseous pneumatic changes of the sacral and iliac bones bilaterally as well as the right pubic and proximal femur bones (Figure 3). Similar pneumatic changes were correlated with the previous MRI of the pelvis (Figure 4). These were stable changes, suggestive of an infectious process in the bones, likely leading to the emphysematous phenomena.

The intra-abdominal collections were drained with mild success. Multiple cultures were grown from various specimens: blood, urine, sputum, fluid collections, wound, multiple skin swabs, and anal abscess aspirate. All were negative except for the anal abscess drainage site, which showed growth of *Escherichia coli* and *Pseudomonas aeruginosa*. In addition, polymerase chain reaction of a sample of the collected intra abdominal fluids was positive for *Bacteroides fragilis*.

Roughly 3 months after initial presentation, the patient deteriorated again. He developed sudden-onset abdominal pain, hypotension, and a drop in haemoglobin level. A large abdominal collection was seen on CT with contrast, which revealed a patent aorta and iliac arteries with no contrast leak. 2 days later, the patient’s periaortic collection had blood density and seemed to have grown in size on CT aortoangiogram, raising concerns of a periaortic haematoma; however, there was no apparent radiological evidence of active arterial extravasation. Moreover, the presence of a new fluid of blood density surrounding the jejunal loops on the left was suggestive of acute haematoma.

Exploratory laparotomy was performed following the patient’s deterioration. An intraoperative diagnosis of an infected aortoiliac bypass graft was made with an accompanying haematoma surrounding the infected graft. The infected aortic Dacron graft was removed with ligation of the aortic and iliac stumps, and left axillo-bifemoral bypass was performed.

**Figure 5.**
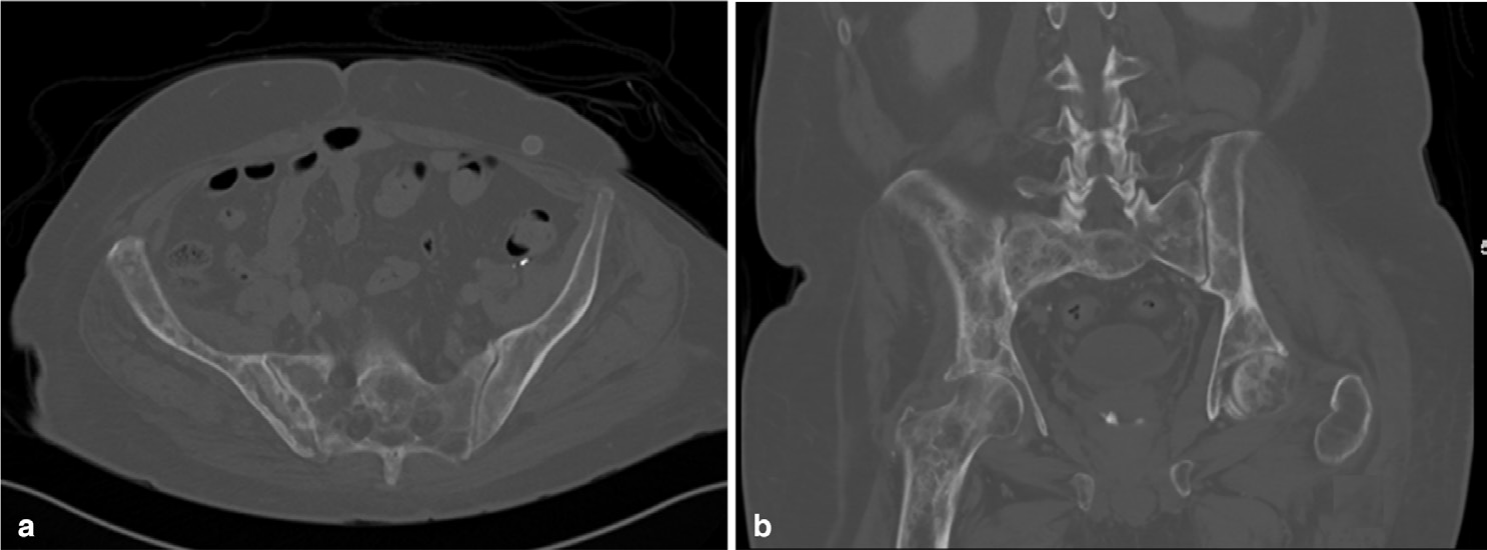
Axial (a) and coronal (b) follow-up CT scan at bone window about 15 months after initial presentation shows resolution of the pelvic and femoral intraosseous gas; however, signs of chronic bony osteonecrotic changes can be visualised.

Upon discharge, the patient was administered 10 more days of oral amoxicillin/clavulanate (1 g BID). He was doing considerably well, with stable vital signs. He had no new complaints and was conscious, alert, and oriented to time, place, and person. In addition, systemic examination was unremarkable. His active issues were a Stage III sacral bed sore, anal fissure managed by laxatives, intra-abdominal fluid collections, and intraosseous gas in the right pelvis and femur. The patient was discharged with the medications he was already prescribed during the hospital stay for his chronic conditions and was provided multiple close follow-up appointments. The patient is currently alive and well. A CT scan (Figure 5) 15 months after his initial presentation revealed resolution of the intraosseous gas; however, chronic osteonecrosis of the bone remains.

## Discussion

EO is intraosseous gas that is often accompanied by signs of an underlying infection.^1,2^ Intraosseous pneumatosis most commonly affects the vertebral bodies, followed by the pelvis and femur.^4^ Although extra axial cases of pneumatosis are highly specific for EO, differentials may include traumatic injury, surgical intervention, neoplasms, lymphangiomatosis of the bone, and osteonecrotic and degenerative diseases of the bone.^3^ In the absence of traumatic fractures and surgical intervention, the occurrence of gas within non-vertebral bones is rather rare.^1–3^ On the other hand, vertebral pneumatosis should direct one towards a non-infectious cause, such as degenerative disease.^5^

Although the pathogenesis of intraosseous pneumatosis is poorly studied, accumulation of nitrogen gas and the vacuum phenomenon as part of a degenerative process have been postulated as possible causes.^6^ Diabetes and neoplastic disease are observed as the most common comorbidities in reported EO cases.^2^ Due to the altered immune status, vascular complications, and autonomic and sensory neuropathy transpiring with uncontrolled hyperglycaemia, poorly managed diabetes leaves the patient at a higher risk of developing severe infections and osteomyelitis.^7^ Our patient’s 15-year history of poorly controlled diabetes mellitus could have acted as a predisposing factor for the patient’s infectious process post-repair.

The route of infection leading to EO could be either haematogenous or contiguous from an intra-abdominal focus or skin or soft tissue organismal colonies.^2,8^ The most commonly implicated pathogens include *E. coli* (28.6%), *Klebsiella pneumoniae* (20%), *Bacteroides* spp (20%), and *Fusobacterium necrophorum* (14.3%).^4^ In the absence of comorbid conditions, the most common microbial organism extracted from specimens was *F. necrophorum*.^2^ In patients with no operative history or an underlying infected collection, a polymicrobial infection is usually suspected.^2^ In reports of post-operative patients, the causative organisms were *Staphylococcus aureus*, *Streptococcus* spp, and *Enterococcus* spp.^1^ In diabetics, *Staphylococcus* and *Enterococcus* spp are often implicated, in addition to stronger microbes such as *P. aeruginosa* and methicillin-resistant *S. aureus*.^7,8^ Moreover, *K. pneumoniae* was reported to be associated with gas formation in the vertebrae and pelvis in a septic patient after multiple abdominal interventions.^9^ In our case, a contiguous spread from the post-aneurysm repair graft abscess is the implicated route of infection. Our patient’s intra-abdominal fluid collections tested positive for *B. fragilis,* although a sample from the affected bone itself was never tested.

The diagnosis of EO is mainly radiological. Recently, Small et al have proposed the ‘pumice stone’ as a radiological sign to identify cases of EO due to the imaging resemblance of EO (irregularly irregular air locules) to the surface appearance of pumice stone (irregularly irregular pores).^10^ They reported it to be present in 96% of cases. They also indicated that, unlike traditional osteomyelitis, most (79%) cases of EO were not associated with cortical destruction of bone.^10^ Although our patient’s imaging studies obviously showed the ‘pumice stone’ sign in the affected bones of the pelvis, there were also signs of cortical destruction.

While MR remains the best imaging modality to identify osteonecrotic lesions and soft tissue changes,^11^ CT is excellent at detecting bony changes and gas. Intraosseous gas can appear on MRI as hypointense foci on *T*_1_- and *T*_2_ weighted images with a corresponding blooming artefact on susceptibility-weighted images (*e.g.* gradient-weighted images).^12^ However, this appearance on MRI is non-specific for gas, as it can be demonstrated with blood products, metallic materials, or sclerotic bone. Hence, CT is more sensitive and specific for the detection of intraosseous gas.^13^

Aggressive management is advised for this rare, often fatal presentation, as mortality rates approach 32% within 7–56 days following a hospital diagnosis of EO.^2^ Surgical intervention by rich debridement with adjunctive organism-specific antibiotic treatment are the modalities of choice in current therapy.

## Conclusion

Although intraosseous gas was rather an incidental finding upon follow-up imaging in our case, it remains an ambiguous, often severe and sudden in onset condition. Knowledge of its clinical radiographic characteristics and associations ought to not only aid physicians in swiftly diagnosing and managing patients, but also shed light on the condition’s vague pathogenesis.

## Learning points

1. Intraosseous pneumatosis occurs mainly in the vertebra, pelvis, and femur.
2. A cause for intraosseous gas must be investigated as the disease can be fatal, regardless of the location.
3. EO should always be suspected in cases of intraosseous gas or osteomyelitis caused by gas-forming organisms; the ‘pumice stone sign’ is considered a pathognomonic sign of EO.
4. Predisposed patients who have undergone interventions, with possible underlying abscesses, are more likely to develop EO.
